# Wireless Sensor Network Coverage Optimization Using a Modified Marine Predator Algorithm

**DOI:** 10.3390/s25010069

**Published:** 2024-12-26

**Authors:** Guohao Wang, Xun Li

**Affiliations:** School of Electronics and Information Engineering, Hebei University of Technology, Tianjin 300401, China; 202231903013@stu.hebut.edu.cn

**Keywords:** wireless sensor network, marine predator algorithm, coverage optimization

## Abstract

To solve the coverage problem caused by the random deployment of wireless sensor network nodes in the forest fire-monitoring system, a modified marine predator algorithm (MMPA) is proposed. Four modifications have been made based on the standard marine predator algorithm (MPA). Firstly, tent mapping is integrated into the initialization step to improve the searching ability of the early stage. Secondly, a hybrid search strategy is used to enhance the ability to search and jump out of local optimum. Thirdly, the golden sine guiding mechanism is applied to accelerate the convergence of the algorithm. Finally, a stage-adjustment strategy is proposed to make the transition of stages more smoothly. Six specific test functions chosen from the CEC2017 function and the benchmark function are used to evaluate the performance of MMPA. It shows that this modified algorithm has good optimization capability and stability compared to MPA, grey wolf optimizer, sine cosine algorithm, and sea horse optimizer. The results of coverage tests show that MMPA has a better uniformity of node distribution compared to MPA. The average coverage rates of MMPA are the highest compared to the commonly used metaheuristic-based algorithms, which are 91.8% in scenario 1, 95.98% in scenario 2, and 93.88% in scenario 3, respectively. This demonstrates the superiority of this proposed algorithm in coverage optimization of the wireless sensor network.

## 1. Introduction

Forests are vital green resources for human beings and play an important role in slowing down climate change [1]. However, forest fires happen frequently nowadays. Thus, how to give early fire warnings in order to preserve the forest has become an important issue to study. The continuous monitoring of the forests using advanced technologies, such as Internet of Things (IoT) [2] and artificial intelligence (AI) [3], could be a feasible solution.

In the field of IoT technology, wireless sensor network (WSN) plays a supportive role [4]. Its sensor nodes disperse within the monitoring region and data transfers via wireless communication [5]. The deployment strategy of sensors and related coverage optimization are important issues to study for WSNs. So far, classical deployment techniques and metaheuristic-based algorithms are the two major methods utilized in the coverage optimization of WSNs.

Classical deployment techniques can be classified into three categories in detail, i.e., computational geometry-based, force-based, and grid-based algorithms [6]. Sensor placement strategies utilizing computational geometry-based algorithms were proposed based on the *k* coverage model using a hexagonal tiling-based approach [7]. But the hexagonal tiling method is only suitable for very few specific coverage scenarios. A virtual force method based on the state of matter was proposed by Vahid using the forced-based algorithm [8]. This algorithm dynamically modified the attracting radius and repulsive force in accordance with the various states of matter, i.e., gas, liquid, and solid, which simulate the behavior of molecules and could ultimately achieve a coverage rate of 83%. Two grid-based algorithms, including grid square coverage versions 1 and 2, were proposed by Ammar with coverage efficiency of 78% and 73%, respectively [9]. However, classical deployment techniques are losing ground in coverage optimization due to the drawbacks of complexity and bad adaptability [10].

Metaheuristic-based algorithms have gained popularity recently due to their high efficiency and good stability [11]. They could be mainly classified into two types, which are evolutionary-based and swarm-based algorithms [12]. Evolutionary-based algorithms, such as genetic algorithm (GA), primarily study evolutionary behavior [13], while swarm-based algorithms, such as sparrow search algorithm (SSA), particle swarm optimization (PSO), and marine predator algorithm (MPA), focus on studying ensemble behavior. For example, an improved SSA was proposed to enhance the ability to jump out of local optima, which could enhance coverage and connectedness while consuming less mobile energy [14]. An improved PSO algorithm was proposed utilizing global optimal value and the Cauchy mutation technique, which could improve the coverage effect in the *k*-coverage problem [15]. MPA was proposed in 2020 and inspired by the predatory behavior in the ocean [16]. Prey and predator have diverse motion modes based on Levy flight and Brownian motion, which could quickly discover the ideal value in complicated issues [17]. However, there are certain disadvantages for the MPA that may affect its performance. For instance, a single trajectory in stages 1 and 3 limits the search ability [18], random initialization leads to uneven distribution [19], and the algorithm easily falls into the local optima [20]. So far, there are few studies related to the usage of improved algorithms of MPA in the field of WSN coverage optimization. An improved MPA proposed by He et al. uses the multi-elite random leading strategy and dynamic inertia weight adjustment strategy to improve the ability of standard MPA to handle the complex problems [21]. A multi-strategy integrated MPA proposed by Wang et al. is utilized in 3D surface WSN coverage optimization [22]. However, none of these studies change the standard three-stage format based on the number of iterations. This article will give a try to modify the original structure of the standard MPA.

In this paper, a modified marine predator algorithm (MMPA) is proposed for coverage optimization of WSN. It utilizes tent mapping for initialization, hybrid search strategy, golden sine search mechanism, and stage adjustment strategy. The optimization capabilities and effectiveness of the proposed algorithm are evaluated by the test functions and coverage tests.

The rest of this paper is organized as follows: The WSN coverage model is presented in Section 2. The standard MPA and proposed MMPA algorithms are described in Section 3. Evaluation results of the proposed algorithm, including test functions and coverage tests, are presented in Section 4. Conclusions are written in Section 5.

## 2. WSN Coverage Model

In this section, the utilized WSN coverage model is presented. Suppose there are *n* randomly distributed sensor nodes in a monitoring area *M*, which could be denoted as *S* = {*S*_1_, *S*_2_, ⋯, *S_j_*, ⋯, *S_n_*}. The symbol *S_j_* represents the *j*th sensor node. Each of the sensor nodes has a fixed sensing radius of *Rs*. The monitoring area *M* is discretized into *L_M_* × *W_M_* grids. The mathematical form of the Euclidean distance is given as follows:

(1) $$d(S_{i},G_{j})=\sqrt{{\left(x_{S}-x_{G}\right)}^{2}+{\left(y_{S}-y_{G}\right)}^{2}},$$

where *d*(*S_i_*, *G_j_*) represents the Euclidean distance between the grid node *G_j_* and the sensor node *S_i_*. The coordinates of *S_i_* and *G_j_* are written as $\left(x_{S},y_{S}\right)$ and $\left(x_{G},y_{G}\right)$, respectively.

The Boolean perception model is adopted in this paper. Its mathematical formulation is written as follows:

(2) $$P(S_{i},G_{j})=\left\{\begin{array}{l} 1,\;\;d(S_{i},G_{j})\leq R_{S}\\ 0,\;\;d(S_{i},G_{j})>R_{S}\; \end{array}\right.,$$

where *P*(*S_i_*, *G_j_*) denotes the probability that the grid node *G_j_* is sensed by the sensor node *S_i_*. When the Euclidean distance *d*(*S_i_*, *G_j_*) is less than or equal to *Rs*, the grid node *G_j_* is considered to be covered by the sensor node *S_j_*. Otherwise, the probability *P*(*S_i_*, *G_j_*) is zero.

Since there are multiple sensor nodes in the monitoring area, the joint sensing probability *P*(*S*, *G_j_*) that a grid node *G_j_* is sensed by all the possible sensor nodes *S* is presented as follows:

(3) $$P(S,G_{j})=1-\prod_{i}^{n}\left(1-P(S_{i},G_{j})\right).$$

The total coverage rate *C_M_* of all sensor nodes *S* in the region *M*, which is used as an objective function in this study, is calculated as follows:

(4) $$C_{M}=\frac{\sum_{i=1}^{L_{M}\times W_{M}}P(S,G_{i})}{L_{M}\times W_{M}}.$$

## 3. Modified Marine Predator Algorithm

In this section, the standard MPA is first introduced, and then an MMPA method with four modifications is proposed based on the standard MPA. Finally, the implementation steps of the MMPA are illustrated.

### 3.1. Standard Marine Predator Algorithm

In the standard MPA, the matrices of the prey *P* and the elite *E* are defined as follows [16]:

(5) $$P={\left[\begin{array}{cccc} X_{1,1} & X_{1,2} & \cdots & X_{1,D}\\ X_{2,1} & X_{2,2} & \cdots & X_{2,D}\\ \vdots & \vdots & \vdots & \vdots \\ X_{N,1} & X_{N,2} & \cdots & X_{N,D} \end{array}\right]}_{N\times D},$$

(6) $$E={\left[\begin{array}{cccc} X_{1,1}^{B} & X_{1,2}^{B} & \cdots & X_{1,D}^{B}\\ X_{2,1}^{B} & X_{2,2}^{B} & \cdots & X_{2,D}^{B}\\ \vdots & \vdots & \vdots & \vdots \\ X_{N,1}^{B} & X_{N,2}^{B} & \cdots & X_{N,D}^{B} \end{array}\right]}_{N\times D},$$

where *N* is the population size and *D* is the dimension. The notation *X_i,j_* in the matrix *P* represents the *j*th dimension of the *i*th prey. The symbol *X^B^* refers to a top predator vector with the best fitness, which is replicated *N* times to construct the elite matrix *E*.

The initial position *X*_0_ of MPA is random, which is determined by the following equation.

(7) $$X_{0}=X_{\min}+rand(X_{\max}-X_{\min}).$$

The symbols *X*_max_ and *X*_min_ represent the upper and lower bounds of the initial individual, respectively. And *rand* is a uniform random number in the range of [0, 1].

After initialization, the optimization process of the standard MPA could be split into three stages according to the relative moving speed of prey and predator.

Stage 1: The prey is moving faster than the predator, which corresponds to the exploration stage. The best course of action for the predator is to remain still while the prey looks for optimization in a wide range. The mathematical model is written as follows:

While *Iter* < *Max_Iter*/3

(8) $$Stepsize_{i}=M_{B}⊗(E_{i}-M_{B}⊗P_{i}),\;i=1,\cdots N,$$

(9) $$P_{i}=P_{i}+\alpha \cdot R_{v}⊗Stepsize_{i}.$$

*Iter* denotes the current number of iterations. *Max_Iter* represents the maximum number of iterations. *Stepsize_i_* stands for the movement step of the individual population. *M_B_* denotes the Brownian motion. The symbol ⊗ represents the product of two vectors. *R_v_* is a vector composed of random numbers between [0, 1]. The notation *α* is a constant with a value of 0.5.

Stage 2: The prey and predator are moving at almost the same speed. This is a transition stage in which the prey is responsible for exploration while the predator is in charge of exploitation. Anyway, the prey moves following the Levy flight strategy and the predator moves using Brownian motion. The total population is equally split into two parts, which are utilized for exploration and exploitation, respectively.

While *Max_Iter*/3 < *Iter* < 2*Max_Iter*/3

(10) $$Stepsize_{i}=M_{L}⊗(E_{i}-M_{L}⊗P_{i}),\;i=1,\cdots N/2,$$

(11) $$P_{i}=P_{i}+\alpha \cdot R_{v}⊗Stepsize_{i},$$

(12) $$Stepsize_{i}=M_{B}⊗(M_{B}⊗E_{i}-P_{i}),\;i=N/2,\cdots N,$$

(13) $$P_{i}=E_{i}+\alpha \cdot \omega ⊗Stepsize_{i}.$$

*M_L_* is a vector of random numbers denoting the Levy flight. The expression for *ω* is given as follows:

(14) $$\omega ={\left(1-\frac{Iter}{Max\_Iter}\right)}^{\left(2\frac{Iter}{Max\_Iter}\right)}.$$

The variable *ω* serves as a controlling factor that regulates the extent of predator movement. As the current number of iterations increases, its value decreases. Thus, the algorithm’s ability to exploit is improved.

Stage 3: In the final stage of the optimization process, the predator is outpacing the prey in terms of velocity. This stage is mainly dominated by exploitation. In the following mathematical equations, a tiny step size is added to the elite position through Levy flight in an attempt to find another position with a higher fitness value.

While *Iter* > 2*Max_Iter*/3

(15) $$Stepsize_{i}=M_{L}⊗(M_{L}⊗E_{i}-P_{i}),\;i=1,\cdots N,$$

(16) $$P_{i}=E_{i}+\alpha \cdot \omega ⊗Stepsize_{i}.$$

Furthermore, environmental issues, such as the fish aggregation devices (FADs) effect and eddy formation, need to be considered since they could lead to behavioral changes in marine predators. The mathematical expression of the FADs effect is written as follows:

(17) $$P_{i}=\left\{\begin{array}{c} P_{i}+\omega [X_{\min}+R_{v}⊗(X_{\max}-X_{\min})]⊗U,\;\mathrm{if}\;r\leq FADs\\ P_{i}+[FADs(1-r)+r](P_{Rand1}-P_{Rand2}),\;\;\;\;\;\mathrm{if}\;r\;>\;FADs \end{array}\right..$$

The denotation *U* is the binary vector 0 or 1. The *FADs* are a constant with a value of 0.2, which represents the probability of the FADs effect. The symbol *r* is a uniform random value between 0 and 1. The subscripts *Rand1* and *Rand2* represent random indices of the prey matrix. In addition, memory saving is used to imitate marine predators remembering the place they have been foraging successfully in MPA.

### 3.2. Initialization Based on Tent Mapping

In the proposed MMPA algorithm, tent mapping is integrated into the initialization step to improve the searching ability of the early stage [23]. The uneven distribution of sensor nodes in the early stage would cause severe problems of coverage overlapping. Using the tent-mapping method could effectively promote the diversity of the population and make the distribution of sensor nodes more uniformly [24].

The expression for tent sequences is written as follows:

(18) $$I_{n+1}=\left\{\begin{array}{cc} \frac{I_{n}}{\varepsilon }, & 0<I_{n}<\varepsilon \\ \frac{1-I_{n}}{1-\varepsilon }, & \varepsilon <I_{n}<1 \end{array}\right..$$

The symbol *I* represents a chaotic sequence in the interval [0, 1]. The symbol *n* represents the *n*th dimension. The symbol *I*_0_ denotes the initial value, which is random. In this study, *ε* is a constant with a value of 0.5. The chaotic matrix can be obtained by constructing the tent sequences (Equation (18)) of *D* dimensions for *N* times. Its expression is written as follows:

(19) $$I={\left\{\begin{array}{cccc} I_{1,1} & I_{1,2} & \cdots & I_{1,D}\\ I_{2,1} & I_{2,2} & \cdots & I_{2,D}\\ \vdots & \vdots & \vdots & \vdots \\ I_{N,1} & I_{N,2} & \cdots & I_{N,D} \end{array}\right\}}_{N\times D}.$$

The initial position *X*_0_ is then obtained by the mapping operation, which is expressed as follows:

(20) $$X_{0}=X_{\min}+I⊗(X_{\max}-X_{\min}).$$

### 3.3. Hybrid Search Strategy

The entire optimization process of the standard MPA is divided into three stages. Since only single-motion style is utilized in stage 1 and 3, i.e., Brownian motion in stage 1 and Levy flight in stage 3, it will lead to inadequate opportunities to explore further in finding the optimal output [18]. In order to solve this problem, the hybrid search strategy, which includes the random movement idea and the usage of the best individual, is introduced to the search process of the proposed MMPA algorithm. The mathematical model of the strategy is given as follows:

(21) $$Stepsize_{i}=2\cdot C\cdot Q-C\cdot A,\;i=1,\cdots N,$$

(22) $$P_{i}=E_{i}+Stepsize_{i}.$$

Here, *Q* is a vector of uniform random numbers, which is in the range of [0, 1]. *A* is a *D*-dimensional vector with all ones. *C* denotes the adaptive control factor and its expression is written as follows:

(23) $$C=(\frac{X_{\max}-X_{\min}}{10})\cdot \exp(-\frac{\pi \cdot Iter}{Max\_Iter}).$$

With the increased current number of iterations, the adaptive control factor *C* could adjust the size of the search range. This hybrid search strategy is utilized in both stage 1 and stage 3 of MMPA but with different constraints. At stage 1, the restriction of Equation (21) is set to be *i* = *N*/2, ⋯*N*. It implies that the first half of the population continues to explore using Equation (9), while the second half explores using Equation (22). The restriction at stage 3 is determined by the mutation probability *R*, which is a random value in [0, 1]. When the value of *R* is greater than 0.8, Equation (22) is used. Otherwise, Equation (16) is chosen. Here, 0.8 is chosen since the optimization accuracy of test functions is the best at this value. Using Equation (22) could realize the mutation in order to raise the capacity of leaving the local optimum. It means that the predator must decide what to do before moving. By using this strategy, there is a significant improvement for the algorithm in the ability to search and escape from local optima.

### 3.4. Golden Sine Guiding Mechanism

In order to improve the convergence ability, the golden sine guiding mechanism derived from the golden sine algorithm is integrated into the proposed MMPA [25]. The golden sine algorithm introduces a golden section coefficient *τ* into the sine algorithm, which could increase the rate of convergence and improve the optimization capacity. As the current number of iterations increases, the golden sine guiding mechanism will focus more on the regions that produce better results than the entire solution space. The optimization process is mathematically expressed as follows:

(24) $$P_{i}^{t+1}=P_{i}^{t}\left|\sin(H_{1})\right|-H_{2}\sin(H_{1})\left|G_{1}O_{i}^{t}-G_{2}P_{i}^{t}\right|.$$

*H*_1_ is a random value ranging from 0 to 2π, while *H*_2_ is a random value in the range of 0 to π. $O_{i}^{t}$ represents the optimal position of the *i*th individual in the *t*th iteration. Both *G*_1_ and *G*_2_ are golden-sine factors, and their expressions are as follows:

(25) $$G_{1}=\omega_{1}(1-\tau )+\omega_{2}\cdot \tau ,$$

(26) $$G_{2}=\omega_{1}\cdot \tau +\omega_{2}(1-\tau ).$$

The notations *ω*_1_ and *ω*_2_ are constants *π* and −*π*, respectively. The golden section coefficient *τ* equals to ($\sqrt{5}-1$)/2.

### 3.5. Stage Adjustment Strategy

Since the hybrid search strategy is used, the function of exploitation and exploration is efficiently combined in stage 1 by utilizing the historical global optimum, and the exploration scope is progressively reduced. In the meantime, stage 1 and stage 3 could be effectively connected by the adaptive control factor *C*. Therefore, stage 2 of the standard MPA loses the original function as a transitional stage since stage 1 already contains the process of transition. So, in the MMPA algorithm, only stage 1 and stage 3 remain while stage 2 is abandoned. This strategy is named as stage adjustment strategy.

### 3.6. Algorithm Steps of MMPA

**Figure 1:**
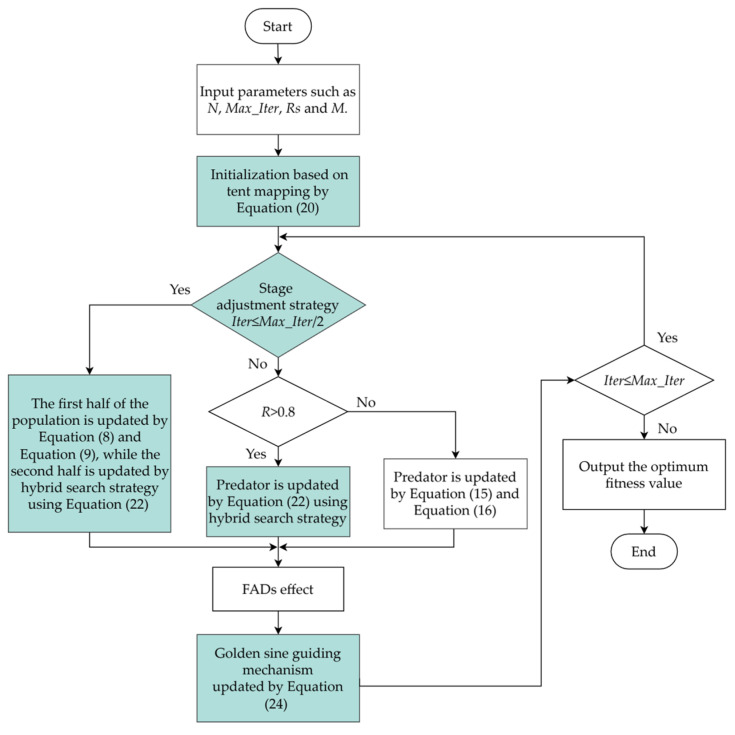
Flow chart of MMPA.

The flow chart of MMPA is given in Figure 1 and its detailed algorithm steps are described as follows:

Step 1: The important algorithm parameters, such as population size *N*, the maximum number of iterations *Max_Iter*, sensing radius *Rs*, and monitoring area size *M* are acquired.

Step 2: After obtaining the tent sequence by tent mapping, the initial population is determined by mapping the tent sequence to the monitoring space using Equation (20). Then, the memory saving is accomplished and the elite matrix *E* is updated.

Step 3: Depending on the current number of iterations, a stage adjustment strategy is applied. When *Iter* is less than or equal to *Max_Iter*/2, both local and global exploration is conducted. The first half of the population is prey, which takes Brownian motion and is updated by Equations (8) and (9). The other half is predator, which is updated by a hybrid search strategy using Equation (22). When *Iter* is greater than *Max_Iter*/2, there are two situations. If the mutation probability *R* is greater than 0.8, the hybrid search strategy is applied to predator using Equation (22). Otherwise, if *R* is less than or equal to 0.8, the predator moves using Levy flight, and Equations (15) and (16) are utilized. And then, the elite matrix *E* needs to be updated and memory saving is implemented.

Step 4: The influence of the FADs effect is considered. The predator performs jump moves using Equation (17). Then, memory saving is accomplished and the elite matrix *E* is updated.

Step 5: The golden sine guiding mechanism is used to expedite the convergence of the population to the optimal solution with Equation (24). Then, memory saving is accomplished and the elite matrix *E* is updated.

Step 6: *Iter* = *Iter* + 1. If the current number of iterations is less than or equal to the maximum value, go back to step 3. Otherwise, go to step 7.

Step 7: Output the optimal solution.

The pseudo-code for the MMPA (Algorithm 1) to optimize coverage of WSN is written as follows:
**Algorithm 1** Pseudo-code of MMPAApply tent mapping to initialize search agents (prey) according to Equation (20), *i* = 1, …, *n* **while** (*Iter* ≤ *Max_Iter*)  Calculate the fitness of *C_M_*, construct the elite matrix and accomplish memory saving  **if** *Iter* ≤ *Max_Iter*/2   For the first half of the population (*i* = 1, …, *n*/2)    Update prey according to Equations (8) and (9)   For the other half of the population (*i* = *n*/2, …, *n*)    Update prey according to Equation (22)  **else if** *Iter* > *Max_Iter/*2   Generate random value R   **if** *R* > 0.8    Update prey according to Equation (22)   **else**    Update prey according to Equations (15) and (16)   **end if**  **end if**  Accomplish memory saving and update the elite matrix  Applying FADs effect according to Equation (17)  Accomplish memory saving and update the elite matrix  Apply golden sine guiding mechanism according to Equation (24)  *Iter* = *Iter* + 1 **end while**

### 3.7. Algorithm Time Complexity Analysis

The time complexity of standard MPA is *O*(*N* × *D* × *Max_Iter*). The time complexity of using tent mapping to initialize the population is *O*(*N* × *D*), so the time complexity is still *O*(*N* × *D* × *Max_Iter*) after this step. Since both the hybrid search strategy and stage adjustment strategy do not adjust the value of *N*, *D,* and *Max_Iter*, the time complexity remains *O*(*N* × *D* × *Max_Iter*). The time complexity of position update using the golden sine guiding mechanism is *O*(*N* × *D* × *Max_Iter*). Therefore, the total time complexity of MMPA is still *O*(*N* × *D* × *Max_Iter*), which is the same as MPA.

## 4. Experimental Results

### 4.1. Experimental Environment

In this study, the MATLAB R2022b software was used to write algorithms on a computer with a 64-bit Windows 10 operating system. This computer was equipped with a CPU of Intel core i5-8300H processor with a main frequency of 2.3 GHz and 16 GB RAM.

### 4.2. Test Functions

Table 1 lists the parameter settings of different algorithms. The proposed MMPA is not only compared with standard MPA [16], but also with other widely used algorithms, such as grey wolf optimizer (GWO) [26], sine cosine algorithm (SCA) [27], and sea horse optimizer (SHO) [28].

Table 2 shows the specific information on the test functions utilized in this study for evaluation. It contains four benchmark test functions (F1, F2, F4, and F5) and two CEC 2017 test functions (F3 and F6). The dimension of the benchmark test functions and the CEC 2017 test functions is set to 30 and 10, respectively. The F1, F2, and F3 test functions are unimodal while the others are multimodal. The optimal values of F3, F4, and F6 are non-zero, while the ideal value for the remaining test functions is zero.

**Figure 2:**
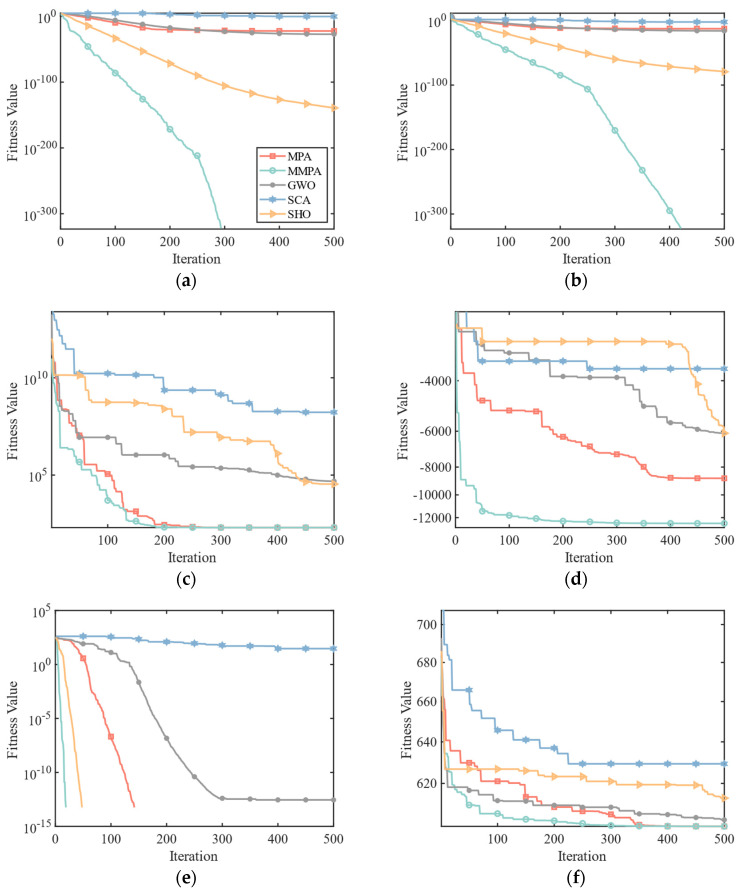
Convergence plot comparison between the proposed MMPA and the other four algorithms on the test functions of (**a**) F1, (**b**) F2, (**c**) F3, (**d**) F4, (**e**) F5, and (**f**) F6.

Figure 2 shows the comparison of convergence plots between the proposed MMPA and the other four metaheuristic-based algorithms, including MPA, GWO, SCA, and SHO, on the six test functions. In order to ensure the fairness of the comparison, the population size *N* of this study is set to 30, and the maximum number of iterations *Max_Iter* is set to 500. The advantage of MMPA is obvious in F1 and F2 test functions. It can be seen that MMPA could find the optimal value zero with fewer iterations while the other algorithms could not jump out of the local optimum. When dealing with the test functions of F3, F5, and F6, both MMPA and other algorithms perform well. However, since the MMPA algorithm employs a hybrid search strategy and stage adjustment strategy, it could attain a more favorable balance between exploration and exploitation, which could find the optimal value in a shorter time. Furthermore, MMPA exhibits superior optimization capabilities when dealing with non-zero optimal solution test functions of F3, F4, and F6. The simulated nonzero optimal solutions are 200, 600, and −12,569.5, respectively, which is the same as the optimal values in Table 2. Table 3 displays the average optimal fitness value (Mean) and the standard deviation (Std) of the optimization results of the proposed MMPA and other four classical algorithms. Each test was run independently for 30 times in order to reduce errors. The MMPA has ideal values for all the test functions except F4, which are the same as the optimization findings shown in Table 2. When it comes to the F4 test function, the calculated average optimal value of MMPA is −12,508.24, which is close to the F4 test function’s optimal value (−12,569.5) shown in Table 2. Furthermore, the Std values of MMPA are the smallest under all test functions compared to the other four algorithms, which implies good algorithm stability. To sum up, the MMPA has a good performance under multiple tests, which confirms the effectiveness of the suggested strategies.

### 4.3. Coverage Test

The efficiency of the suggested MMPA algorithm in coverage optimization will be evaluated in this section. The specific parameters designed for three coverage scenarios are shown in Table 4. The parameters, such as area size *M*, number of nodes *n*, and node-sensing radius *Rs*, will be taken into account. The number of populations *N* is set to 30 for all the simulations.

**Figure 3:**
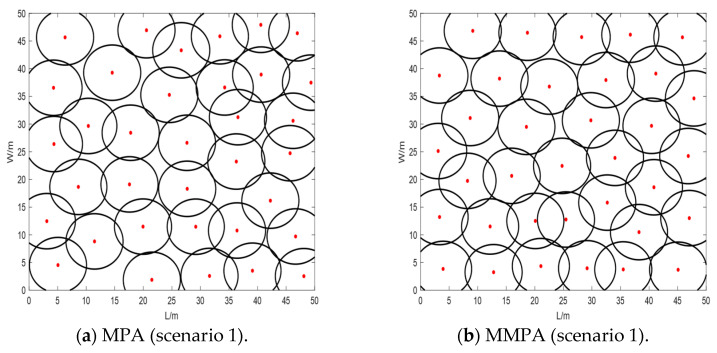

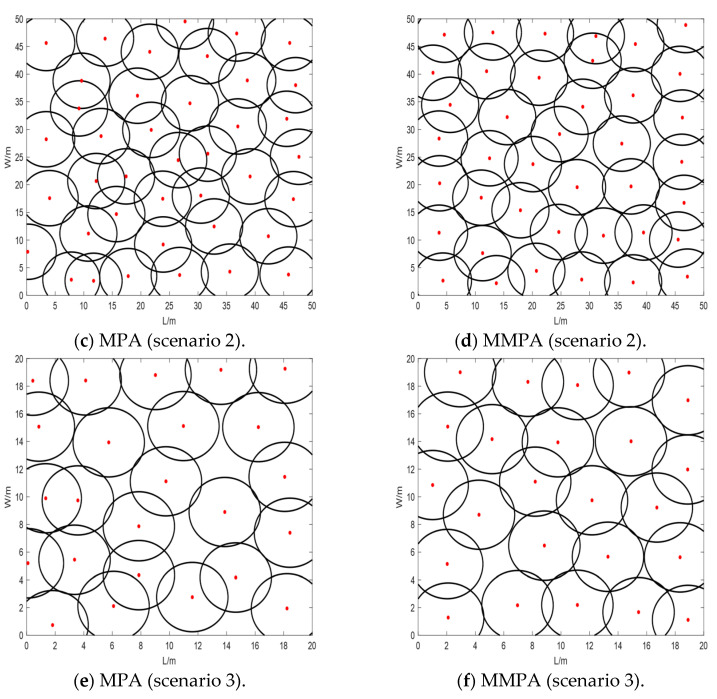
Distribution maps of sensor nodes at (**a**) scenario 1 using MPA, (**b**) scenario 1 using MMPA, (**c**) scenario 2 using MPA, (**d**) scenario 2 using MMPA, (**e**) scenario 3 using MPA, and (**f**) scenario 3 using MMPA, respectively.

Figure 3 shows the distribution maps of sensor nodes in three different scenarios using both MPA and MMPA algorithms. It can be seen that the distribution maps using MPA have certain overlapped coverage areas for all three scenarios. This overlapped area is located in the upper right corner of scenario 1 (Figure 3a), in the middle part of scenario 2 (Figure 3b), and in the bottom left corner of scenario 3 (Figure 3c), respectively. In contrast, the sensor node distribution maps of MMPA are more uniform with no large overlapped area for all three coverage scenarios. The coverage rates for all the conditions are compared as follows: The initial coverage rate *C_M_* is 68.49% in scenario 1 where 35 sensor nodes are used for calculation. The coverage rate is 89.12% when the standard MPA is applied. It is increased to 91.97% when using the suggested MMPA algorithm, which is 2.85% higher than that of MPA. In the case of scenario 2, the initial coverage rate is 74.97%, with 40 sensor nodes. The coverage rate of MMPA is increased to 96.50%, which is 3.07% greater than that of MPA (93.43%). The initial coverage rate is 70.48% for scenario 3 with 24 nodes. The coverage rate of MMPA is 94.56% in this scenario, which is 3.40% greater than that of MPA (91.16%).

In order to further evaluate the effectiveness of MMPA in coverage optimization, the coverage rate is calculated 30 times for each scenario and its average value is presented in Table 5. The average coverage rate of MMPA is the highest for all the scenarios compared to other algorithms, such as MPA, improved sparrow search algorithm (ISSA) [29], self-adaptive multi-strategy artificial bee colony (SaMABC) [30], modified specular reflection optimization algorithm (MRSA) [31], an algorithm combining the genetic algorithm and reinforced whale optimization algorithm (GARWOA) [32], and enhanced sparrow search algorithm (ESSA) [33]. This verifies the excellent ability of the proposed MMPA to handle coverage optimization problems.

## 5. Conclusions

In order to solve the insufficient coverage problem of WSN in the forest-fire monitoring system, a new algorithm named MMPA is proposed based on the standard MPA. Four modifications have been made to the original algorithm. Firstly, tent mapping is utilized to improve the quality of the initial population in order to obtain a better search ability at the early stage. Secondly, a hybrid search strategy is used to enhance the ability to search and jump out of local optimum. Thirdly, the golden sine guiding mechanism is applied to accelerate the convergence of the algorithm. Finally, the stage adjustment strategy is proposed to make the transition more smoothly. The performance of MMPA is evaluated by both test functions and coverage tests. It shows that the MMPA has good optimization capability and stability by the results of test functions. Compared to MPA and other metaheuristic-based algorithms, MMPA has the highest average coverage rates in all three different scenarios, which are 91.8% in scenario 1, 95.98% in scenario 2, and 93.88% in scenario 3, respectively. And at the same time, the node distribution of MMPA is relatively uniform. The obtained results verify the superiority of MMPA in the coverage optimization of WSN.

## Figures and Tables

**Table 1:** Parameter settings of different algorithms.

Algorithms	Parameters
MMPA	*FADs* = 0.2, *P* = 0.5, *ε* = 0.5
MPA	*FADs* = 0.2, *P* = 0.5
GWO	Adaptive value *a* linearly decreased from 2 to 0
SCA	Constant *A* = 2
SHO	*u* = 0.05, *v* = 0.05

**Table 2:** Parameter of the test functions.

Function No.	Name	Domain	Optimum
F1	Sphere	[−100, 100]	0
F2	Schwefel 2.22	[−10, 10]	0
F3	Shifted and Rotated Sum of Different Power Function	[−100, 100]	200
F4	Schwefel 2.26	[−500, 500]	−12,569.5
F5	Rastrigin	[−5.12, 5.12]	0
F6	Shifted and Rotated Expanded Scaffer’s F6 Function	[−100, 100]	600

**Table 3:** Optimization result comparison of different algorithms on six test functions.

No.	Index	MMPA	MPA	GWO	SCA	SHO
F1	Mean	0	4.21 × 10^−23^	8.21 × 10^−28^	4.68	1.49 × 10^−139^
	Std	0	3.52 × 10^−23^	8.50 × 10^−28^	6.96	7.88 × 10^−139^
F2	Mean	0	2.88 × 10^−13^	8.11 × 10^−17^	0.02	2.33 × 10^−77^
	Std	0	3.16 × 10^−13^	5.38 × 10^−17^	0.02	1.14 × 10^−76^
F3	Mean	200.00	200	1.31 × 10^8^	1.08 × 10^8^	5.57 × 1
	Std	0	0	6.40 × 10^8^	2.57 × 10^8^	1.69 × 10^9^
F4	Mean	−12,508.24	−8884.94	−5994.72	−3757.87	−6131.08
	Std	230.21	387.49	734.99	213.67	462.97
F5	Mean	0	0	2.46	44.01	0
	Std	0	0	3.03	31.10	0
F6	Mean	600	600.02	601.52	622.10	617.27
	Std	0	0.05	1.93	4.05	5.13

**Table 4:** Parameter settings of coverage test.

Parameters	Scenario 1	Scenario 2	Scenario 3
Area size	50 m × 50 m	50 m × 50 m,	20 m × 20 m
Number of nodes	35	40	24
Node-sensing radius	5 m	5 m	2.5 m
Communication radius	10 m	10 m	5 m
Maximum iteration	500	500	500

**Table 5:** Comparison of average coverage rate.

Coverage Scenario	Algorithm	Average Coverage Rate
Scenario 1	random deployment	68.54%
	MPA	89.56%
	MMPA	91.80%
	ISSA [29]	91.48%
Scenario 2	random deployment	73.97%
	MPA	93.83%
	MMPA	95.98%
	SaMABC [30]	94.98%
	MSRA [31]	95.08%
	GARWOA [32]	95.73%
	ESSA [33]	94.80%
Scenario 3	random deployment	70.88%
	MPA	91.30%
	MMPA	93.88%

## Data Availability

The original contributions presented in this study are included; further reasonable inquiries can be directed to the author.

## References

[1] Verkerk P.J., Costanza R., Hetemäki L., Kubiszewski I., Leskinen P., Nabuurs G.J., Potocnik J., Palahí M. (2020). Climate-smart forestry: The missing link. For. Policy Econ..

[2] Gokhale P., Bhat O., Bhat S. (2018). Introduction to IOT. Int. Adv. Res. J. Sci. Eng. Technol..

[3] Zhai X., Chu X., Chai C.S., Jong M.S.Y., Istenic A., Spector M., Liu J., Yuan J., Li Y. (2021). A Review of Artificial Intelligence (AI) in Education from 2010 to 2020. Complexity.

[4] Soliman H., Sudan K., Mishra A. A smart forest-fire early detection sensory system: Another approach of utilizing wireless sensor and neural networks. Proceedings of the 2020 IEEE Sensors.

[5] Singh O., Rishiwal V., Chaudhry R., Yadav M. (2021). Multi-objective optimization in WSN: Opportunities and challenges. Wirel. Pers. Commun..

[6] Farsi M., Elhosseini M.A., Badawy M., Ali H.A., Eldin H.Z. (2019). Deployment techniques in wireless sensor networks, coverage and connectivity: A survey. IEEE Access.

[7] Ammari H.M. (2023). A computational geometry-based approach for planar k-coverage in wireless sensor networks. ACM T. Sensor Network.

[8] Kiani V., Soltani A. (2022). Improved Virtual Force Algorithm based on the States of Matter for Improving Coverage of Mobile Wireless Sensor Networks. Int. J. Ind. Electron. Control Optim..

[9] Hawbani A., Wang X., Husaini N., Karmoshi S. (2014). Grid coverage algorithm & analysis for wireless sensor networks. Netw. Protoc. Algorithms.

[10] Adu-Manu K.S., Engmann F., Sarfo-Kantanka G. (2022). WSN protocols and security challenges for environmental monitoring applications: A survey. J. Sensors.

[11] Osamy W., Khedr A.M., Salim A., Al Ali A.I., El-Sawy A.A. (2022). Coverage, deployment and localization challenges in wireless sensor networks based on artificial intelligence techniques: A review. IEEE Access.

[12] Boussaïd I., Lepagnot J., Siarry P. (2013). A survey on optimization metaheuristics. Inform. Sci..

[13] Singh P., Kottath R. (2021). An ensemble approach to meta-heuristic algorithms: Comparative analysis and its applications. Comput. Ind. Eng..

[14] Yao Y., Liao H., Liu M., Yang X. (2023). Coverage Optimization Strategy for 3-D Wireless Sensor Networks Based on Improved Sparrow Search Algorithm. IEEE Sens. J..

[15] Ling H., Zhu T., He W., Luo H., Wang Q., Jiang Y. (2020). Coverage optimization of sensors under multiple constraints using the improved PSO algorithm. Math. Probl. Eng..

[16] Faramarzi A., Heidarinejad M., Mirjalili S., Gandomi A.H. (2020). Marine Predators Algorithm: A nature-inspired metaheuristic. Expert Syst. Appl..

[17] Al-Betar M.A., Awadallah M.A., Makhadmeh S.N., Alyasseri Z.A.A., Al-Naymat G., Mirjalili S. (2023). Marine predators algorithm: A review. Arch. Comput. Method. E..

[18] Jin Z., Jiang J., Kong Z., Pan C., Ruan X. (2023). A novel coverage optimization scheme based on enhanced marine predator algorithm for urban sensing systems. IEEE Sens. J..

[19] Zhang C., He Z., Li Q., Chen Y., Chen S., Nie X. (2023). An adaptive marine predator algorithm based optimization method for hood lightweight design. J. Comput..

[20] Hu G., Zhu X., Wei G., Chang C.T. (2021). An improved marine predators algorithm for shape optimization of developable Ball surfaces. Eng. Appl. Artif. Intell..

[21] He Q., Lan Z., Zhang D., Yang L., Luo S. (2022). Improved marine predator algorithm for wireless sensor network coverage optimization problem. Sustainability.

[22] Wang Z., Xiao H., Yang S., Wang J., Mahmoodi S. (2022). Multistrategy integrated marine predator algorithm applied to 3D surface WSN coverage optimization. Wirel. Commun. Mob. Com..

[23] Li Y., Han M., Guo Q. (2020). Modified whale optimization algorithm based on tent chaotic mapping and its application in structural optimization. KSCE J. Civ. Eng..

[24] Chen L., Song N., Ma Y. (2023). Harris hawks optimization based on global cross-variation and tent mapping. J. Supercomput..

[25] Tanyildizi E., Demir G. (2017). Golden sine algorithm: A novel math-inspired algorithm. Adv. Electr. Comput. En..

[26] Mirjalili S., Mirjalili S.M., Lewis A. (2014). Grey wolf optimizer. Adv. Eng. Softw..

[27] Mirjalili S. (2016). SCA: A sine cosine algorithm for solving optimization problems. Knowl.-Based Syst..

[28] Zhao S., Zhang T., Ma S., Wang M. (2023). Sea-horse optimizer: A novel nature-inspired meta-heuristic for global optimization problems. Appl. Intell..

[29] Wang J., Zhu D., Ding Z., Gong Y. WSN Coverage Optimization based on Improved Sparrow Search Algorithm. Proceedings of the 2023 15th International Conference on Advanced Computational Intelligence (ICACI).

[30] Wang J., Liu Y., Rao S., Zhou X., Hu J. (2023). A novel self-adaptive multi-strategy artificial bee colony algorithm for coverage optimization in wireless sensor networks. Ad Hoc Netw..

[31] Ma B., Liu Y., Han H., Lv P., Zhou Q., Hu Y. (2023). Coverage optimization of WSNs based on modified specular reflection optimization algorithm. Transducer Microsyst. Technol..

[32] Sun S., Chen Y., Dong L. (2024). An optimization method for wireless sensor networks coverage based on genetic algorithm and reinforced whale algorithm. Math Biosci. Eng..

[33] Gao S., Li Z., Feng F. (2024). WSN coverage optimization based on ISSA. Intern. Things Tech..

